# RURAL HOSPICE SOCIAL WORK: SUPPORTING PRACTITIONERS IN END-OF-LIFE WORK WITH LIMITED RESOURCES

**DOI:** 10.1093/geroni/igac059.163

**Published:** 2022-12-20

**Authors:** Brandi Felderhoff, Angela Alvarado, Valerie Alvarez

**Affiliations:** Texas Woman's University, Denton, Texas, United States; Kindred at Home, Hospice Division, Dallas, Texas, United States; Perimeter Behavioral Hospital, Arlington, Texas, United States

## Abstract

Rural areas and the agencies, including hospice, that serve them face immense challenges in terms of accessibility, and service delivery. Small hospices face an enigmatic combination of higher operating costs and lower reimbursement payments, forcing higher caseloads on staff, and straining already limited available resources. Multiple cost benefit studies indicate that Medicare hospice reimbursement rules are well suited to the expense structure of large volume hospices, usually in urban, population dense areas; however, it is not clear that they apply as abundantly to smaller volume, rural hospices. This study sought to garner a deeper understanding of the roles and challenges required for rural hospice social work practice. Individual interviews with 9 rural hospice social workers across organizations in Texas and New Mexico were conducted. A maximum variation sampling technique was used to purposefully sample social workers from hospice agencies in areas deemed as rural by the Association of Rural Communities in Texas, with fewer than 200,000 occupants in their counties. Using emergent thematic analysis, key themes materialized including the challenge of dual relationships, required tasks beyond the scope of practice, issues of autonomy, and meeting them (patients/families) where they are at. Results demonstrate the complexities of rural hospice social work practice, the culture of rural communities, and the need for research into evidence-based intervention strategies specific to rural hospice social work, that will guide practitioners through navigating these challenging conditions.

